# Tertiary lymphoid structures as local perpetuators of organ-specific immune injury: implication for lupus nephritis

**DOI:** 10.3389/fimmu.2023.1204777

**Published:** 2023-10-31

**Authors:** Meiying Wang, Snehin Rajkumar, Yupeng Lai, Xingjiao Liu, Jing He, Tatsuya Ishikawa, Dhiraj Nallapothula, Ram Raj Singh

**Affiliations:** ^1^ Department of Rheumatology and Immunology, Shenzhen Second People’s Hospital, The First Affiliated Hospital of Shenzhen University, Shenzhen, China; ^2^ Peking University Shenzhen Hosiptal, Shenzhen, China; ^3^ Autoimmunity and Tolerance Laboratory, Division of Rheumatology, Department of Medicine, David Geffen School of Medicine at University of California Los Angeles (UCLA), Los Angeles, CA, United States; ^4^ Department of Nephrology, Peking University Shenzhen Hospital, Shenzhen, China; ^5^ Department of Pathology and Laboratory Medicine, David Geffen School of Medicine at UCLA, Los Angeles, CA, United States; ^6^ Molecular Toxicology Interdepartmental Program, David Geffen School of Medicine at UCLA, Los Angeles, CA, United States; ^7^ Jonsson Comprehensive Cancer Center, David Geffen School of Medicine at UCLA, Los Angeles, CA, United States

**Keywords:** autoimmune disease, ectopic lymphoid tissue, lupus nephritis, systemic lupus erythematosus, tertiary lymphoid structure

## Abstract

In response to inflammatory stimuli in conditions such as autoimmune disorders, infections and cancers, immune cells organize in nonlymphoid tissues, which resemble secondary lymphoid organs. Such immune cell clusters are called tertiary lymphoid structures (TLS). Here, we describe the potential role of TLS in the pathogenesis of autoimmune disease, focusing on lupus nephritis, a condition that incurs major morbidity and mortality. In the kidneys of patients and animals with lupus nephritis, the presence of immune cell aggregates with similar cell composition, structure, and gene signature as lymph nodes and of lymphoid tissue-inducer and -organizer cells, along with evidence of communication between stromal and immune cells are indicative of the formation of TLS. TLS formation in kidneys affected by lupus may be instigated by local increases in lymphorganogenic chemokines such as CXCL13, and in molecules associated with leukocyte migration and vascularization. Importantly, the presence of TLS in kidneys is associated with severe tubulointerstitial inflammation, higher disease activity and chronicity indices, and poor response to treatment in patients with lupus nephritis. TLS may contribute to the pathogenesis of lupus nephritis by increasing local IFN-I production, facilitating the recruitment and supporting survival of autoreactive B cells, maintaining local production of systemic autoantibodies such as anti-dsDNA and anti-Sm/RNP autoantibodies, and initiating epitope spreading to local autoantigens. Resolution of TLS, along with improvement in lupus, by treating animals with soluble BAFF receptor, docosahexaenoic acid, complement inhibitor C4BP(**β**-), S1P1 receptor modulator Cenerimod, dexamethasone, and anti-CXCL13 further emphasizes a role of TLS in the pathogenesis of lupus. However, the mechanisms underlying TLS formation and their roles in the pathogenesis of lupus nephritis are not fully comprehended. Furthermore, the lack of non-invasive methods to visualize/quantify TLS in kidneys is also a major hurdle; however, recent success in visualizing TLS in lupus-prone mice by photon emission computed tomography provides hope for early detection and manipulation of TLS.

## 1 Introduction

Tertiary lymphoid structures (TLS), also known as tertiary lymphoid organs, ectopic lymphoid neogenesis, or ectopic lymphoid tissues, are organized clusters of immune cells that form in non-lymphoid tissues after birth. TLS are neither stable structures nor present in embryonic life, but instead can be induced by chronic inflammatory stimuli and tissue injury (1, 2) in autoimmune diseases such as Sjogren syndrome, rheumatoid arthritis, systemic lupus erythematosus (SLE), and myositis (3–5), allograft rejection (2, 6), chronic infections (5), and cancers (7–9).

Impaired tolerance to self-antigens via various mechanisms marks the early step in the pathogenesis of autoimmune diseases (10, 11), resulting in the initial activation of autoreactive cells. These activated autoimmune cells may then infiltrate the target organs, where the persistent exposure to antigens in chronically inflamed environments keeps them activated. Such chronically activated immune cells can serve as a substitute for lymphoid tissue inducer (LTi) cells and initiate the formation of TLS in tissues. TLS may execute tissue-specific immune responses, thereby providing communications between immune cells and local resident cells (12). This process typically aids in the clearance or neutralization of pathogens through the local induction of plasma cells that generate specific antibodies (13). TLS often resolve after successful antigen clearance or upon resolution of inflammation. However, TLS resolution might not happen in the context of persistent antigen presentation in autoimmune-prone backgrounds, thereby enabling the local induction and expansion of autoreactive T and B cells, which may cause increased autoantibody production and contribute to local pathology as in SLE.

SLE is a highly heterogeneous disease, with different patients exhibiting different manifestations. Kidneys may be involved in up to 50% of adults and up to 80% of children with SLE (14, 15). Kidney disease in SLE, called lupus nephritis (LN), continues to be a major contributor of morbidity and mortality in SLE (16, 17). Current treatments for LN may cause systemic immune suppression with many adverse effects. Hence, understanding local mechanisms of disease in kidneys may open new avenues to develop organ-targeted treatments for LN, thereby avoiding systemic toxicity. In this article, we will review the current understanding of TLS as it pertains to LN.

## 2 Secondary lymphoid organs

TLS share similarities with secondary lymphoid organs (SLO) with regard to their structure and function (18–21). Understanding the development of SLO may provide clues to mechanisms underlying TLS formation. SLO consist of lymph nodes, spleen, tonsils, Peyer’s patches, and mucosa-associated lymphoid tissues distributed throughout the body. These structures play a role in initiating and organizing adaptive immune responses by facilitating the interaction between antigens and immune cells. The development and formation of SLO, particularly lymph nodes and Peyer’s patches, involve CD34^+^ hematopoietic stem cells and TNF-related activation-induced cytokine (TRANCE^+^) stromal cells, as well as cytokines, chemokines, adhesion molecules, and specialized vasculature (22). SLO formation, which occurs during embryogenesis or in the first few weeks after birth, involves hematopoietic lymphoid tissue inducer (LTi) cells, a subtype of innate lymphoid cells (CD45^+^CD4^+^CD3^−^) that express RORγt and Id2. The process also involves the interactions of LTi cells with TRANCE and CXC-chemokine ligand 13 (CXCL13) through their receptors, TRANCE-R and CXCR5, respectively (23). Once LTi cells aggregate, their function is sustained by IL-7. This induces the expression of lymphotoxin α1β2 which binds to the lymphotoxin β receptor expressed on mesenchymal lymphoid tissue organizer (LTo) cells. This binding promotes LTo cells to express adhesion molecules, including vascular cell adhesion molecule 1 (VCAM1), intercellular adhesion molecule 1 (ICAM1), mucosal addressin cell-adhesion molecule 1 (MAdCAM1), and peripheral node addressin (PNAd), and to generate lymphoid chemokines, such as CC-chemokine ligand 19 (CCL19), CCL21, and CXCL13. Then, the arrangement of lymphocytes expressing CCR7 and CXCR5 receptors, which contributes to the formation of T cell and B cell zones within SLO, is facilitated by follicular reticular cells that express CCL19 and/or CCL21 and follicular dendritic cells (FDCs) that express CXCL13 (24).

Germinal center (GC) formation in SLO is regulated by antigen-driven interactions between B cells and follicular T helper (Tfh) or follicular T regulatory cells (Tfr). Tfh cells support the GC reaction through their expression of CD40L that engages CD40 on GC B cells, activates the NF-κB pathway, and triggers B cell proliferation. Tfh cells can also upregulate the Tfr–associated transcription factor Foxp3, which dampens Tfh cells’ expression of CD40L and IL-21 and contributes to GC extinction (25). The transcription factor BCL6 is essential to the differentiation of Tfh cells and the formation of GCs. In the absence of T cell–expressed BCL6, B cell responses to protein antigens do not occur and GCs fail to form (26). GCs are crucial for generating high-affinity antibodies against pathogens. GC B cells are positively selected for clonal expansion based on affinity of their cell surface receptors for an antigen – high affinity for an antigen favors differentiation of GC B cells into proliferative plasmablast cells. Some of these plasmablasts subsequently enter the quiescent plasma cells compartment, ultimately resulting in the generation of high-affinity, long-lived plasma cells and memory B cells within the GC (27). Somatic hypermutation has the potential to generate self-reactive immunoglobulins and humoral autoimmunity, whereas negative selection in GC proper entails elimination by apoptosis of GC B cells that acquire self-reactivity through somatic hypermutation (25).

The distinct microenvironmental niches within SLO that harbor different sets of innate and adaptive immune cell populations are formed by the structural scaffold of follicular reticular cells, which also generate conduit networks for cell communication (28). In addition, specialized blood vessels known as high endothelial venules (HEVs) play a role in guiding lymphocytes to SLO. They facilitate the extravasation of lymphocytes from the bloodstream, a necessary step for the initiation of adaptive immune responses in lymphoid tissues (29–31). HEVs are critical components of the immune system that facilitate lymphocyte recirculation and immune surveillance, playing a role in recognizing and responding to pathogens as well as in chronic inflammatory diseases (32).

Patients with SLE exhibit many abnormalities of SLO, including follicular hyperplasia, coagulative necrosis, diverse histological lesions, and increased 18-F-fluoro-2-deoxy-D-glucose (^18^F-FDG) uptake (33, 34). A higher ^18^F-FDG accumulation was also found in organs with higher immune cell compositions such as the lymph nodes, spleen, and thymus as compared to organs like the brain, liver, and lungs in NZB/NZW F1 mouse model of lupus (35). These observations suggest that immune cells in SLO in humans and animals with lupus are in an active state. Although the exact pathogenic contributions of these abnormalities are not fully understood, SLO abnormalities may contribute to autoimmune pathogenesis in multiple ways including initial breakdown of tolerance and activation of autoreactive T and B cells (10, 11, 36). For example, tissue-resident dendritic cells that carry local antigens to tissue-draining lymph nodes to induce tolerance do not migrate well to lymph nodes in lupus-prone mice, thus contributing to loss of tolerance and inflammation (37, 38).

## 3 Tertiary lymphoid structures: initiation and formation

**Figure 1:**
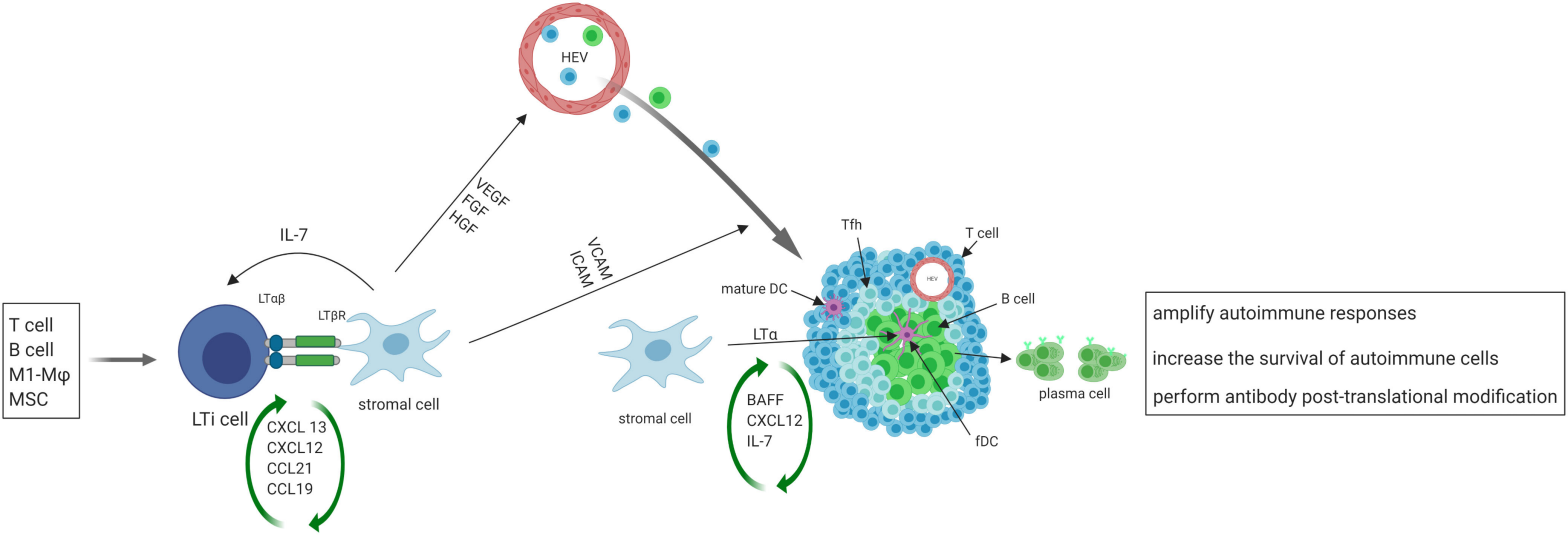
The initiation, formation and function of TLS. Activated immune cells produce CXCL13 and IL-7, recruit LTi cells to the site of inflammation. LTi cells secrete lymphotoxin (LT) αβ, which binds to lymphotoxin β receptor (LTβR) expressed on local stromal cells, inducing the production of chemokines such as CXCL13, CXCL12, CCL21, and CCL19. These chemokines then trigger the recruitment of lymphocytes, which initiate the development of TLS. The interaction of LTβ and LTβR also leads to the secretion of VEGF, FGF and hepatocyte growth factor (HGF) by stromal cells, as well as the secretion of vascular cell adhesion molecule 1 (VCAM1) and ICAM1. VEGF, FGF, and HGF play a crucial role in promoting the development of high endothelial venules (HEVs), while ICAM and VCAM expressed on local stromal cells facilitate the recruitment of T and B cells from nearby HEVs and promote their eventual organization into TLS. Some local stromal cells differentiate into FDCs upon stimulation with LTα, and FDCs aid in the development and sustenance of TLS by producing chemokines and providing a cellular network that facilitates B cell migration. A subset of CD4+ T cells polarizes into T follicular helper (Tfh, CXCR5^hi^PD-1^hi^ICOS^hi^) cells that promote B cell activation and antibody production. TLS facilitate the process of affinity maturation and differentiation of naïve B cells into germinal center B cells and plasma cells that produce antibodies.

After the initial activation in SLO, activated immune cells may migrate to the target organs where the persistent exposure to antigens in chronically inflamed environments may keep immune cells activated. Such activated cells in tissues may serve as primary regulators of local TLS formation, analogous to the role of classical LTi cells in SLO. Activated immune cells produce CXCL13 and IL-7, and recruit LTi cells to the site of inflammation (39) in a positive feedback loop. T helper 17 (Th17) cells (40), B cells (41), and M1-polarized macrophages (42) have been shown to possess the ability to replace LTi cells and initiate TLS formation in various pathological conditions (**Figure 1**).

On the other hand, stromal cells play the same role in TLS formation as classical LTo in SLO. At sites of inflammation, LTi cells aggregate and release lymphotoxin αβ, which binds to the lymphotoxin β receptor expressed on stromal cells residing in the area (43). The resident stromal cells express the classical LTo cell phenotype and respond to the accumulation of LTi cells by inducing the production of homeostatic chemokines such as CXCL13, CXCL12, CCL21, and CCL19. These chemokines recruit lymphocytes to the site of inflammation and initiate TLS formation (18–20, 44). Upon activation of the lymphotoxin β–lymphotoxin β receptor signaling pathway in stromal cells and surrogate LTi cells, the secretion of several growth factors such as vascular endothelial growth factor (VEGF), fibroblast growth factor (FGF), and hepatocyte growth factor are induced (45). Additionally, adhesion molecules such as VCAM1 and intercellular adhesion molecule 1 (ICAM1) are also secreted (46). VEGF, FGF, and hepatocyte growth factor are critical for promoting the development of HEVs. Additionally, the presence of ICAM and VCAM on stromal cells in the vicinity aids in the recruitment of T and B cells from nearby HEVs and promotes the organization of these cells into TLS (47).

Following the organization of TLS by cytokines/chemokines, some local stromal cells differentiate into FDCs upon stimulation with lymphotoxin α. FDCs play a role in supporting the development and maintenance of TLS by producing chemokines and providing a cellular network for B cell migration (13). A subset of CD4^+^ T cells polarizes into T follicular helper (Tfh, CXCR5^hi^PD-1^hi^ICOS^hi^) cells that promote B cell activation and antibody production (48). In addition to CD4^+^ T follicular helper (Tfh) cells, T cell compartments also contain CD8^+^ cytotoxic T cells, CD4^+^ T helper 1 (Th1) cells, and regulatory T cells (Tregs) (49). TLS display similar organization to SLO, where B cells aggregate around FDCs expressing the CD21 antigen, and T cells areas contain dendritic cells expressing the marker MIDC-8 (50). Subsequently, TLS facilitate the process of affinity maturation and differentiation of naïve B cells into GC B cells and plasma cells that produce antibodies (51). As in SLO, follicular reticular cells form a dense network to anchor TLS at the site of chronic inflammation (1). At the same time, vasculature comprised of HEVs and lymphatic vessels forms the surrounding compartments to facilitate ingress and egress of immune cells.

Taken together, activated immune cells can function as primary regulators of TLS formation, substituting for LTi cells. Resident stromal cells function as LTo cells to stimulate the production of cytokines and chemokines, creating a positive feedback loop that induces the formation of GCs by lymphoid cells. The development of TLS can be divided into three sequential stages. The first stage, known as the ‘immature’ or ‘early’ TLS, is composed of dense lymphocytic aggregates with mostly T cells and fibroblasts, but no FDCs and without segregated T and B cell zones. The second stage, referred to as the ‘premature’ or ‘primary follicle-like’ TLS, contains FDCs and small B cell follicle but lacks GC reaction. The third stage, considered as the ‘mature’ or ‘secondary follicle-like’ TLS, is characterized by a GC that appears as well-organized aggregates of T and B cells with segregated T and B cell zones, presence of FDC networks, development of HEVs, and specialized lymphoid fibroblasts (49, 52).

TLS formation relies on inducible inflammatory triggers, such as CXCL13 and CCL19, as well as lymphorganogenic cytokines like lymphotoxin αβ. Transcription factor BCL-6, which is essential for Tfh differentiation (52), was overexpressed in TLS in skin lesions (53). Importantly, reducing B cell activating factor (BAFF) decreased TLS formation in an inducible model of LN (54), suggesting a role of BAFF in TLS formation. The processes of B cell selection, activation, affinity maturation, isotype switching, and plasma cell formation can occur in GCs of TLS in a manner similar to that in GCs of SLO (55). Studies in patients and animal models of autoimmune diseases, such as autoimmune diabetes, myasthenia gravis and Wegener’s granulomatosis, have demonstrated the process of selection and affinity maturation of B cells in TLS. For example, granulomas of upper respiratory tract in patients with Wegener’s granulomatosis contain clusters of B lymphocytes, dendritic cells and plasma cells with evidence of initial selection and affinity maturation of B cells, which is potentially driven by self-antigen proteinase-3 (56). B cell selection, activation, affinity maturation, isotypic switching and plasma cell formation have also been demonstrated in TLS associated with tumors, resulting in an “in situ” anti-tumor antibody production (57). Thus, mature TLS with GCs are active sites of generation of autoantibodies and antibodies against tumor antigens. Additionally, molecular and cellular interaction in the TLS promotes the local inflammatory response in the organs targeted by autoimmune diseases, as described below for kidneys in LN.

## 4 TLS in LN

TLS are found in autoimmune diseases’ target tissues such as kidneys (lupus), salivary glands (Sjögren’s syndrome), and synovial joints (rheumatoid arthritis) (58). TLS have been reported in aged human kidneys as well as in kidneys from patients and animals with various kidney diseases including IgA nephropathy, renal allograft rejection, and pyelonephritis (59, 60). In many conditions, the extent and stage of TLS appear to correlate with disease severity (59, 60). These observations suggest that TLS formation may be a common pathological process, which may progress with disease such as LN.

### 4.1 Evidence for the formation of TLS in LN

The first demonstration of TLS in lupus likely came from an induced model, where lipogranulomas picked from the peritoneal wall of BALB/cJ mice injected with hydrocarbon oil TMPD exhibited organized structures with HEV and discrete areas of B and CD4^+^ T cells, and activated DCs, and lymphoid chemokines (61). The TLS in these lipogranulomas recapitulated many functional characteristics of SLO (62). Subsequent studies in spontaneous and induced models, including NZB/NZW F1, MRL-lpr, BXSB, anti-nucleosome IgG passive transfer, silica instillation and TLR8-knockout mice, demonstrated the formation of TLS (**Table 1**). TLS in lupus mouse kidneys had similar cell composition, structure, and gene signature as lymph nodes (50), and therefore may function as a kidney-specific type of lymph node. A comparison with human LN gene expression revealed similar up-regulated genes as observed during the development of murine LN and TLS (50). In animal models, TLS formation was associated with the development of systemic autoimmune disease (65) and with local autoantibody production (66).

**Table 1:** Evidence supporting the formation of TLS in LN: Animal model studies.

Study	LN Model	Detection of TLS	Findings	Comment
Nacionales, et al, 2006 (61)	TMPD-induced LN in female BALB/cJ mice	Lipogranulomas picked from the peritoneal wall of mice, stained with H&E, and for immune cells	Lipogranulomas are organized structures with MECA-79^+^ (peripheral lymph node addressin) HEV, discrete areas of B and T cells and activated DCs, and lymphoid chemokines.	TMPD granulomas are ectopic lymphoid tissue, the first evidence of ELT/TLS in lupus
Nacionales, et al, 2009 (62)	TMPD-induced lupus in BALB/cJ	Lipogranulomas analyzed with IHC, RT-PCRs, class-switched transcripts, ELISPOT, ELISA	Lipogranulomas contained diverse B cell repertoires and local GC-like reactions, class-switched anti-RNP Ab-producing cells and T cell-dependent somatic hypermutation.	TLS induced by TMPD recapitulates many functional characteristics of SLO.
Kang, et al, 2017 (54)	AID^-/-^MRL-lpr mice injected with anti-nucleosome IgG	H&E and immunostained kidney; TLS counted by the size of cell clusters and organization	6-fold increase in compartmentalized clusters of B, T, and myeloid cells and FDCs in the tubulointerstitial area in mice with induced lupus.	Renal TLS formation induced in a passive transfer model of LN
Dorraji, et al, 2020 (50)	NZB/NZW F1 females with anti-DNA Ab	Kidney sections stained with hematoxylin, RNA isolation, and immunohistochemistry	TLS resembling interconnected networks with T/B cell zones, DCs, and GCs. Gene profiles of kidneys with LN showed upregulated genes related to TLS formation.	Kidney TLS have a similar cell composition, structure, and gene signature as lymph nodes.
Masum, et al, 2020 (63)	MRL-lpr and BXSB/MpJ-Yaa (Yaa) mice	H&E, PAS, and immunostaining of kidney slices; electron microscopy; and transcriptomics	Vascular and perivascular structures showed lymphoid tissue organization with markers for T, B and plasma cells, macrophage, HEV, FDCs, and lymphorganogenic chemokines.	VALT in kidneys is a unique TLS.
Chauhan, et al, 2021 (64)	NZB/NZW F1, with intranasally instilled silica	Analyses of BALF and lung tissue on days 1, 7, 14, 21 and 28 after silica exposure	Neutrophils/macrophages/lymphocytes recruited into alveoli, cell death, elevated cytokines, chemokines, BAFF, and IFN-I (d7), followed by the emergence in the lung of organized T (d14) and B cells (d21), with features of ELT.	Temporal evolution of TLS after exposure to an environmental trigger in autoimmune mice
Wang, et al., 2021 (65)	TLR8^-/-^ model-LN/Sjogren’s disease	TLR8^-/-^ C57BL/6 mice; salivary gland and lung histology and IF	TLR8^-/-^ mice develop TLS characterized by B/T aggregates, HEV formation, and the presence of DC in salivary glands.	TLS formation associated with systemic autoimmunity
Fee, et al, 2022 (66)	Female BXSB, silica instillation	H&E or PAS and immunostaining of lung tissue.	Increased TLS in the lungs; anti-myeloperoxidase and anti-dsDNA autoAbs detected in BALF and lung tissue.	Increased TLS associated with local autoAb production

Ab, antibody; BAFF, B cell activation factor; BALF, broncho-alveolar lavage fluid; d, day; DC, dendritic cells; dsDNA, double-stranded DNA; ELT, ectopic lymphoid tissue; FDC, follicular dendritic cells; FFPE, formalin-fixed paraffin embedded; GC, germinal center; H&E, hematoxylin and eosin; HEV, high endothelial venule; IF, immunofluorescence; IHC, immunohistochemistry; i.p., intraperitoneal; LN, lupus nephritis; SLO, secondary lymphoid organ; Tg, transgenic; TMPD, 2,6,10,14-tetramethylpentadecane, also known as pristane; VALT, vasculature-associated lymphoid tissue.

A timed exposure to an environmental trigger allowed the investigators to assess a temporal evolution of TLS in lupus. Silica instillation in NZB/NZW F1 mice induced the recruitment of neutrophils, macrophages and lymphocytes into alveoli, with local cell death, elevated cytokines, chemokines, BAFF, and IFN-I by day 7, followed by the emergence in the lung of organized T (day 14) and B cells (day 21), with features of TLS (64).

Soon after the description of TLS in an animal model of lupus (61), Steinmetz and colleagues identified four organizational levels of intrarenal aggregates in patients with LN, ranging from scattered B cells to highly structured clusters with central FDCs (67) (**Table 2**). Majority of the organized tubulointerstitial infiltrates in LN kidneys were well-circumscribed T:B cell aggregates, also considered as class II ectopic lymphoid tissue, which were identified in 46% to 57% of LN biopsies (68–70). Most of the remaining organized infiltrates were described as class I ectopic lymphoid tissue and less than 10% had class III that were GCs containing FDCs with B cell clonal expansion and somatic hypermutation. GCs contained CD138^–^CD20^+^ centroblasts, whereas T:B aggregates had CD138^+^CD20^low/2^ plasmablasts (68). Most patients with class IV LN had TLS in kidney biopsies, up to 94% of cases in one study. Thus, animal model and human studies have demonstrated the formation of TLS in LN.

**Table 2:** Evidence supporting the formation of TLS in LN: Human studies.

Study	Patient population	Detection of TLS	Findings	Comment
Steinmetz, et al, 2008 (67)	32 patients with LN and 16 with ANCA associated nephritis	Biopsies subjected to immunohistochemistry and Real-time PCR analysis.	Four organizational levels of intrarenal aggregates in LN patients, ranging from scattered B cells to highly structured clusters with central FDCs.	TLS was discovered as a new classification of B cell clusters.
Chang, et al, 2011 (68)	68 LN, 81% black, class II (n=3), III (22), IV (33) and V (10).	Kidney biopsy sections stained for CD3, CD20, CD45, MUM1, CD138, CD21, Ki-67, BAFF, FDC.	52% biopsies with tubulointerstitial infiltrate organized into well-circumscribed T:B cell aggregates (46%) or GCs containing FDCs (6%). The remaining 46% had scattered lymphocytic infiltrates (46%).	More than 2/3^rd^ of organized structures vs. 18% of scattered infiltrates had proliferating T/B cells, suggesting a functional lymphoid structure.
Shen, et al, 2012 (69)	192 LN, Chinese, LN class I (7), II (5), III (13), IV (71), V (43), III/IV+V (52)	Intrarenal B cells analyzed in biopsies by IHC for CD20, CD3, and CD21.	62% biopsies had intrarenal B cells in the renal interstitium, which associated with class IV LN and with higher activity and chronicity indices. No association with anti-dsDNA, anti-Sm, C3/C4.	Formation of intrarenal interstitial B-cell aggregates is a common response in LN
He, et al, 2016 (70)	89 LN, Chinese, class III (21), IV (53), V (15); all LN treated with corticosteroids + immunosuppressants	Renal biopsy stained for CD3, CD20, and CD21 by IHC.	78% biopsies had ELTs (57% with type II ELT, 20% with type I ELT). 50/53 (94%) LN class IV had ELTs. Intrarenal B cells associated with a longer disease course and poor clinical response.	Intrarenal B cells associated ELTs are common in patients with severe proliferative LN.

ANCA, anti-neutrophil cytoplasmic antibody; ELT, ectopic lymphoid tissue; FDC, follicular dendritic cells; IHC, immunohistochemistry; LN, lupus nephritis; TLS, tertiary lymphoid structure.

### 4.2 Potential mechanisms underlying TLS formation in LN

In kidney biopsies from patients with LN, CXCL13 expressed in cells with dendritic morphology in areas of infiltration with B cells that expressed the cognate receptor CXCR5 (67). Furthermore, the presence of TLS in human kidney biopsies correlated with increased B cell hyperactivity and serum levels of CXCL13 (70). Subsequently, transcriptomic studies of LN kidneys in MRL/lpr and BXSB models showed increased *cxcl13*/*cxcr5* along with upregulated expression signature associated with TLS formation (63), suggesting a role of CXCL13/CXCR5 in TLS formation in LN. In animal studies, BAFF, TLR7 signaling, and Fli-1 have also been suggested to play a role in TLS formation (54, 65, 71). These and other molecular and cellular mechanisms associated with TLS formation in lupus are described in **Table 3**. In kidney injury models, genetic deletion or antibody neutralization suggested a role of IL-17A in TLS formation in kidneys (74). Among cellular mechanisms, mesenchymal stem cells have been suggested to act as LTi cells in kidneys through interaction with T cells, thus inducing TLS formation in LN (72). Other studies suggest a possible role of innate lymphoid cells type 3 in perivascular tissues and impaired tissue-resident dendritic cells in TLS formation (37, 73). Identification of molecules associated with TLS formation (**Table 4**) and their possible roles in TLS formation and LN pathogenesis would be critical for therapeutic targeting of TLS.

**Table 3:** Potential mechanisms that may promote TLS formation in lupus.

Study	Patients/Model	Study design	Findings	Comment
Steinmetz, et al, 2008 (67)	32 patients with LN	Lymphoid chemokine BCA-1 (CXCL13) and its receptor CXCR5 detected by IHC/real time PCR	In regions of B cell infiltration, increased BCA-1 expressed in cells of a dendritic-like morphology and most B cells expressed CXCR5	BCA-1/CXCR5 may play role in B cell infiltration into the kidney.
He, et al, 2016 (70)	89 LN and 25 non-LN SLE	Serum CXCL13 by ELISA, and renal biopsy staining for B cells.	LN patients with kidney TLS had more serum CXCL13 than those without. Serum CXCL13 levels correlated with the number of B cells in kidney biopsies	CXCL13 might be involved in renal TLS formation.
Kang, et al, 2017 (54)	AID-/-MRL/lpr passive transfer (anti-nucleosome IgG) model	BAFF-secreting cells enumerated by ELISpot in isolated kidney cells. Treating mice with BR3-Fc to reduce BAFF.	Increased BAFF production in the kidneys during LN development. Reducing BAFF *in vivo* prevented the formation of TLS and LN	BAFF induces TLS during LN
Dorraji, et al, 2018 (72)	NZB/NZW F1 mice kidneys, and human MSCs	IF staining of kidneys from mice, and *in vitro* culture of human MSC with T cells.	MSCs detected in TLS in NZB/NZW kidneys. Stimulated human MSCs increased the expression of CCL19, VCAM1, ICAM1, TNF-α, and IL-1β, and induced T cell proliferation	Tissue-specific or migratory MSCs could play roles as LTo cells in initiating kidney TLS.
Masum, et al, 2020 (63)	MRL-lpr and BXSB/MpJ-Yaa (Yaa) mice	Transcriptomic analysis of kidney tissue	Increased *Cxcl13* and CXCR5, upregulated expression signatures associated with lymphoid tissue formation, leukocyte migration, HEV forming, and adhesion molecules	Identified molecules associated with TLS formation in kidneys
Wang, et al., 2021 (65)	TLR8-ko model of LN and Sjogren’s disease	Double TLR7/8-deficient C57BL/6 mice; histology and IF of salivary gland and lung tissues	TLS formation is abrogated in double TLR7/8-deficient mice	Suggests a role of TLR7 signaling in TLS formation
Li, et al, 2023 (73)	MRL-lpr mice	ILC detected in kidneys by microscopy and transcriptomics	Increased ILC3 in perivascular ELTs; ILC3 cells promoted differentiation of B cells into plasma cells	Potential role of ILC3 cells in TLS formation/function
Sato, et al, 2023 (71)	MRL-lpr, wild-type and Fli-1^+/−^	Serum and renal CXCL13, renal infiltrate, TLS, and LN	Reduced renal CXCL13^+^ immune cells in kidneys in Fli-1^+/−^ MRL/lpr	Fli-1 may regulate TLS formation in LN kidneys via CXCL13
Eriksson and Singh, 2008 (37)	MRL/lpr mice	Staining of skin and lymph node for DC	Skin-resident DCs do not migrate to the draining lymph nodes, accumulate as clusters in skin, and attract other immune cells, which mimics ELT	A possible role of impaired DC migration in TLS

BR3-Fc, soluble BAFF receptor fused to the Fc portion of mouse IgG1; DC, dendritic cells; ELT, ectopic lymphoid tissue; Fli-1, Friend leukemia virus integration 1; IF, immunofluorescence; IHC, immunohistochemistry; ILC3, group 3 innate lymphoid cells; ko, knockout; LTo, lymphoid tissue organizer cells; MSC, mesenchymal stem-like cells; VALT, vasculature-associated lymphoid tissue.

**Table 4:** Molecules associated with TLS formation.

Molecule	Functions
CXCL13, IL-7	Recruit LTi cells to the site of inflammation
CXCL13, CXCL12, CCL21, CCL19	Recruit lymphocytes and initiate TLS formation
VEGF	Vascular formation
FGF	Form a dense network to anchor TLS
ICAM, VCAM	Recruitment of T and B cells
BAFF	Induce TLS

### 4.3 Possible contributions of TLS to LN

Although the principal lesion within the kidney in LN is glomerulonephritis, tubulointerstitial inflammation is common. Severity of tubulointerstitial disease on renal biopsy, rather than severity of glomerular disease, predicts progression to renal failure (75, 76). Tubulointerstitial lesions commonly have TLS containing T:B cell aggregates, plasmablast foci, and GCs (68). These TLS are sites of *in situ* antigen-driven selection of B cells. Thus, human LN appears to arise from both systemic and *in situ* autoimmune responses, with the latter more closely associated with a poor prognosis (6, 7, 12). Consistently, TLS was associated with higher disease activity and chronicity indices and poor treatment response in LN (68–70). TLS was also associated with the progression of kidney damage in other models of kidney injury (74).

In lupus-prone mice, anti-dsDNA antibody positive animals developed TLS in their kidneys, and when they became proteinuric, a large and organized TLS with GC-like structures formed within the kidney (50, 54, 72). These animals had a higher ex vivo FDG accumulation in the kidney of anti-dsDNA antibody positive group compared to antibody-negative mice, which is consistent with the presence of large population of metabolic active or activated immune cells (35). Thus, TLS may contribute to LN in multiple ways (**Table 5**), such as by being a local source of activated immune cells (35), local IFN-I production (61), maintaining systemic autoantibody production (66, 78), site for intermolecular epitope spreading (80, 81) to local autoantigens such as to locally overexpressed vimentin (79), and facilitating tubulointerstitial inflammation (63, 68).

**Table 5:** Evidence supporting potential contributions of TLS to LN.

Study	Model/Study Population	Study design	Findings	Comments
Nacionales et al, 2006 (61)	TMPD-induced LN in female BALB/cJ mice	Lipogranulomas analyzed by RT-PCR and qPCR	Increased expression of IFN-inducible genes (*IRF-7*, *Mx1*, *IP-10*, and *ISG15* in lipogranulomas	TLS as a site for production of IFN-I that plays a role in lupus pathogenesis
Chang et al, 2011 (68)	68 LN, 25% on low-dose prednisone	Kidney biopsy histology and immunohistochemistry	Presence of GCs or T:B aggregates in tubulointerstitial area was associated with tubular basement membrane ICs (60%), and with severe interstitial inflammation (94%)	TLS may contribute to pathogenesis of tubulointerstitial inflammation in LN.
Liarski et al, 2014 (77)	Kidney biopsies from LN or renal transplant patients	Cell distance mapping to identify Tfh cells in LN biopsies.	The presence of follicular T cell-like CD4 T cells in kidney biopsy samples was associated with lower estimated glomerular filtration rate in patients with LN	A potential pathogenic contribution of TLS in LN
Weinstein et al, 2013 (78)	TLS transplant model	Lipogranuloma from TMPD-injected into naïve recipient mice	Recipients of transplanted lipogranulomas produced anti-U1A autoAbs derived exclusively from the donor	TLS plasma cells as a source of maintaining lupus autoAb (anti-Sm/RNP) production
Kinloch et al, 2014 (79)	8 LN patients, kidney biopsies	Laser captured or flow-sorted CD38^+^ or Ki-67^+^ B cells from interstitial infiltrates cloned and tested for antigen reactivity.	10 of the 25 Abs produced by B cells isolated from tubulointerstitial lesions bound vimentin. Serum anti-vimentin levels associated with severe tubulointerstitial LN.	*In situ* local antigen-driven immune response in tubulointerstitial ELTs likely feeds forward to worsen LN.
Masum et al, 2020 (63)	MRL-lpr mice	Kidney slices immunostaining and transcriptomics	Transcriptional profile and intracellular interaction also demonstrated antigen presentation, lymphocyte activity, clonal expansion, follicular, and GC activity in VALT.	VALT/TLS size was correlated with glomerular and tubulointerstitial lesions
Dorraji et al, 2021 (35)	NZB/NZW F1 mice	PET/^18^F-FDG combined with CT of kidneys	Higher ex vivo FDG accumulation in the kidney of anti-dsDNA Ab positive mice vs. Ab-negative mice	As a source of metabolically active or activated immune cells
Fee et al, 2022 (66)	Tg ABM autoAb M7 in C57BL/6 background, post silica instillation	Immunostained lung tissue; autoAbs in BALF and lung tissue	Increased lung TLS in silica exposed Tg mice; specific Tg autoAb produced by lung B cells	TLS facilitates the recruitment and supports survival of autoreactive Tg B cells, and systemic autoimmunity

^18^F-FDG, 18-F-fluoro-2-deoxy-D-glucose; Ab, antibody; ABM, Anti-basement membrane autoantibody; BALF, broncho-alveolar lavage fluid; CT, computed tomography; ELT, ectopic lymphoid tissue; ICs, immune complexes; PET, positron emission tomography; Tg, transgenic; Tfh, T follicular helper cells; TLS, tertiary lymphoid structure; VALT, vascular-associated lymphoid structure.

### 4.4 Potential mechanism whereby TLS might promote LN development.

Animal and human studies suggest several mechanisms by which TLS might contribute to lupus disease (**Table 6**). First, TLS may promote high-affinity autoantibody production. In lupus mice, B cell proliferation, somatic hypermutation, and class switch recombination are found within TLS (62). In human studies, most kidney biopsies from LN patients had well-defined T:B cell aggregates or GCs containing FDCs within tubulointerstitial infiltrates, which correlated with intrarenal B cell clonal expansion and ongoing somatic hypermutation (68). Dendritic cells in lupus mice infiltrate the kidney, present antigens, and locally amplify inflammation signals (84), thus promoting TLS. FDCs express Fcγ receptors and complement receptors, allowing for the binding of antigen-antibody complexes and presentation of antigens to B cells. This leads to the selection of B cells that produce high-affinity antibodies (85). Furthermore, TLS GCs harbor proliferating B lymphocytes expressing activation-induced deaminase, a key enzyme in somatic hypermutation and class switch recombination, resulting in the generation of high-affinity, class-switched antibodies (86).

**Table 6:** Summary of possible mechanisms by which TLS might contribute to LN.

Mechanism	References
Persistence of autoimmune responses at renal site	(82)
Survival of autoreactive lymphocyte clones	(78)
Post-translational modifications of antibodies	(83)
Intrarenal B cell clonal expansion and ongoing somatic hypermutation	(68, 63)
Antigen presentation	(63, 67)
Epitope spreading to local autoantigens	(79)

Second, TLS may contribute to LN development by promoting the longevity of autoreactive lymphocytes. In the TMPD-SLE model, autoreactive memory B cells and anti-Sm/RNP autoantibody-producing plasma cells home to TLS (78), which may allow them to escape from normal immune censoring mechanisms in this location. TLS can provide survival signals for incoming lymphocytes and differentiated long-lived plasma cells through the secretion of IL-7, CXCL12, and BAFF (87). In support of the latter, transgenic overexpression in mice of BAFF, a critical B cell survival factor, induces autoimmune nephritis (88). An inflammatory environment could also promote mesenchymal stem cells’ production of lymphocyte survival cytokines, IL-7 and CXCL12, which can increase T cell recruitment and CD4^+^ T cell proliferation and differentiation in kidney-TLS in lupus mice (72).

Third, the site of antibody synthesis and the local microenvironment can influence the post-translational modifications of immunoglobulins (87). Thus, TLS’ effect on post-translational modifications, such as glycosylation and sialylation, may influence the function of antibody, and their pathogenic potential in the context of autoimmune disease. For example, altered glycosylation of IgG in the kidneys of MRL-lpr mice has been associated with LN (89), whereas the transfer of sialylated autoantigen-reactive IgG antibody into lupus-prone FcγRIIB-deficient mice attenuated LN (90). Although, the data directly demonstrating the regulation of post-translational modification of autoantibody by TLS are lacking in LN patients, there have been several reports on the association between altered glycosylation and inflammation in patients with SLE (91).

Fourth, a human study analyzed the antigen specificity of antibodies produced by B cells isolated by laser-capture or flow sorting from tubulointerstitial lesions in kidney biopsies from LN patients (79). Ten of the 25 antibodies thus identified bound vimentin which was overexpressed in these kidneys. Thus, *in situ* local antigen-driven immune response in tubulointerstitial TLS may lead to autoimmune spreading, which might lead to worsening of LN.

## 5 GC vs. extrafollicular responses in the context of TLS in LN

The kidney infiltrates in patients with LN may range from scattered B and T cells to the fully organized TLS with GCs containing FDCs (67, 68, 77) (**Table 2**). Among LN kidney biopsies with organized infiltrates, about 10% exhibited TLS with typical features of GCs containing FDCs with B cell clonal expansion and somatic hypermutation (68), about 50% of patients had well-circumscribed T:B cell aggregates, and the remaining patients had dense lymphocytic aggregates without segregated T and B cell zone and without FDCs (68–70). The latter have been suggested to be ‘immature’ or ‘early’ TLS or class I ectopic lymphoid tissue, while others have argued that these organized kidney infiltrates are distinct from TLS (92). Chronic autoimmune responses in these organized lympho-myeloid infiltrates have been suggested to be extrafollicular (92, 93). Animal studies have also suggested that spontaneous or induced responses in TLS can, but do not have to, include GCs (62). In fact, extensive intraclonal somatic V region diversification was observed in a persistent non-canonical extrafollicular response in lupus-prone mice (94). The same autoreactive B cell clonotype proliferating and mutating extrafollicularly and in GCs was found in lupus-prone NZM.2410 mice (95). Furthermore, the mutation rate in autoreactive extrafollicular responses in MRL/lpr mice was similar to that seen in peak GCs (96). Somatic hypermutation and isotype switching were also observed in synovial lymphoid infiltrates without a GC-like structure of rheumatoid arthritis patients (97). In these and other studies in autoimmune diseases’ target tissues, at least some of the microdissected B cell clusters that yielded mutated V region sequences did not resemble GC structures and were most likely an extrafollicular-like response (92).

There is some evidence to suggest that certain infections and immune inflammatory states favor the GC or the extrafollicular response (92). It has been suggested that the extrafollicular response may occur in the context of severe infections with high pathogen burden (92). Corollary to this, the abundant antigenic target of the immune response, such as DNA or DNA-containing immune complexes as in SLE, may trigger an extrafollicular response, whereas the localized antigen, such as insulin as in type I diabetes, may trigger a GC response. The degree of inflammation and cytokine response may also dictate the GC vs. extrafollicular response. It is also likely that autoimmune responses exist along a spectrum from GC- to extrafollicular-dominant, and may even evolve over time with the severity of disease in an individual.

In SLE, B cells in the GC are influenced by the expression of Absent in Melanoma 2 (AIM2). Reduced AIM2 expression can decrease B cells in the GC and alleviate lupus symptoms (98), highlighting the role of GC in lupus disease. However, blocking the GC pathway by knocking out the transcription factor *Bcl6* in GC B cells did not protect animals from anti-dsDNA antibody production, plasma cell output, and immune complex deposition in glomeruli in the R848 model (99). Understanding these different autoimmune scenarios might help to stratify patients within a given autoimmune disease (100), which might help guide the selection of targeted therapies.

## 6 Clinical implications and therapeutic targeting of TLS

Persistence of TLS in autoimmune conditions is associated with a poor prognosis and with a failure to respond to B cell-depleting therapies (3, 101). Therefore, identifying mechanisms underlying the formation and persistence of TLS could present new therapeutic options for autoimmunity. For example, treatment of mice with BR3-Fc (soluble BAFF receptor fused to the Fc portion of mouse IgG1) reduced BAFF levels and prevented the formation of TLS in kidneys and development of LN in an inducible model of SLE (54). In recent clinical trials, adding belimumab that blocks BAFF to standard therapy in people with active LN improved renal function, decreased flare rate and allowed glucocorticoid withdrawal (102, 103). It remains to be determined whether belimumab reduced TLS in the kidneys of patients with LN.

In animal studies, several treatments have been shown to reduce TLS and autoimmune disease (**Table 7**). For example, treating MRL-lpr mice with a blocking antibody against CXCL13 that plays a role in TLS formation improves neuropsychiatric lupus (108). Treatment with a classical pathway complement inhibitor C4BP(**β**-) or with the selective S1P1 receptor modulator, Cenerimod, also reduced TLS and autoimmune disease in lupus-prone mice (105, 106). Additionally, dexamethasone was highly effective in preventing TLS formation in MR/lpr mice (63). These observations further emphasize the role of TLS in the development and progression of lupus and open avenues for modulating TLS as a treatment option.

**Table 7:** Potential therapeutic targeting of TLS.

Study	Animal Model	Intervention	Outcome	Implications
Kang et al, 2017 (54)	AID^-/-^MRL-lpr mice, with a passive transfer (anti-nucleosome IgG) model	Treating mice with BR3-Fc (soluble BAFF receptor fused to the Fc of mouse IgG1)	Treatment reduces BAFF, which prevented the formation of TLS and development of LN.	Reducing BAFF reduces TLS and LN
Bates et al, 2018 (104)	NZB/NZW F1, with silica induced accelerated GN	Feeding DHA, an ω-3 polyunsaturated fatty acid, prior to silica instillation	Delayed TLS formation in lungs, systemic anti-dsDNA antibody production, and GN	Preventing TLS formation associated with reduced LN
Luque et al, 2020 (105)	NZB/NZW F1 and MRL-lpr mice	C4BP(**β**-), a classical pathway complement inhibitor, i.p.	Reduced renal TLS formation, serum anti-dsDNA, and LN, improved survival	Reduced TLS associated with decreased LN
Gerossier et al, 2021 (106)	MRL-lpr, spontaneous chronic sialadenitis	Cenerimod, a selective S1P1 receptor modulator	Reduced T cells and proliferating plasma cells within salivary gland TLS, resulting in diminished disease-relevant autoAbs within the salivary glands	Cenerimod may reduce TLS and disease in kidneys and other organs
Masum et al, 2020 (63)	MRL-lpr female mice	Dexamethasone oral and i.p.	Treated mice had almost no or reduced size VALT and reduced *Cxcl9* and *Cxcl13* expression in kidneys compared to the control group	Ablation of VALT/TLS may be a mechanism of action of corticosteroids in LN
Pestka et al, 2021 (107)	NZB/NZW F1, with silica induced accelerated GN	Feeding DHA after onset of TLS formation	Suppressed/delayed recruitment of T/B cells to the lung, lung TLS formation, systemic anti-dsDNA antibody production, and GN	Established TLS can be suppressed, which was associated with reduced LN
Huang et al, 2021 (108)	MRL-lpr model of NP-lupus, with TLS in brain and increased CXCL13	Anti-CXCL13 or control antibody injected i.p. or intracerebroventricularly	Improved cognitive function and depression-like behavior in the treated mice, regardless of administration method	CXCL13 blockade as a potential treatment to reduce TLS and lupus
Torres et al, 2022 (109)	huCD20 focal DTH-TLS model of multiple sclerosis	B cell depletion with s.c. or i.v. ofatumumab and ocrelizumab	Reduced extent of glial activation as well as the number of B- and T-cells in the lesion	B cell depletion may target TLS
Heine et al, 2022 (110)	NZB/NZW F1, with silica induced accelerated GN	Prednisone, low, moderate or high doses, given in feed	Mice fed with moderate doses of prednisone had reduced TLS in lungs, serum ANAs, and GN	TLS as a potential target of corticosteroid therapy in LN

DHA, docosahexaenoic acid; DTH, delayed-type hypersensitivity; GN, glomerulonephritis; huCD20, humanized CD20 transgenic; i.p., intraperitoneal; i.v., intravenous; NP, neuropsychiatric; s.c., subcutaneous; TLS, tertiary lymphoid structure; VALT, vasculature-associated lymphoid tissue.

TLS can also serve as a prognostic marker, as the presence of TLS in the kidneys of lupus-prone mice and patients with LN (68, 111) is often associated with poor prognosis and increased autoantibody production (3). Furthermore, analysis of TLS densities in LN biopsies, their cell composition and cytokine/chemokine secretions, and the antibody produced by plasma cells educated in TLS might help in predicting therapeutic responses to immune modulating drugs. Further work is needed to determine if the persistence of TLS correlates with a poor response to treatment in LN.

## 7 Challenges and future directions for TLS in LN

Many questions remain regarding TLS as well as relative contributions of TLS among the cascade of pathogenic events that lead to LN. TLS studies thus far have used variable evaluation strategies. A comprehensive analysis of TLS phenotypes, density and spatial distribution using a standardized, reproducible, quantitative approach is needed. For example, a study in human melanoma used 7-color multiplex immunostaining of whole tissue sections from 103 human melanoma samples to characterize TLS phenotypes along the expression of established TLS-defining molecular and cellular components (112). Such integrated qualitative, quantitative, and spatial analysis of TLS at different stages of LN using a large human sample size can inform the relative contributions of TLS in LN.

The lack of non-invasive methods to visualize/quantify TLS in kidneys is a major hurdle in using TLS in the clinical assessment. However, Dorraji and colleagues were able to visualize TLS in the pancreas of lupus-prone, anti-dsDNA antibody positive, NZB/NZW F1 mice by single photon emission computed tomography (SPECT) using ^99m^TC labeled Albumin Nanocoll (35). Further advancement in SPECT and *in vivo* imaging technologies may make it possible to detect and longitudinally monitor TLS in LN.

Further research is needed to understand the composition and anatomy of TLS and roles of different cell types in TLS function. TLS develop in near proximity to large veins and arteries and contain lymph vessels and micro capillaries (50, 72). The larger renal drainage lymphatics in mice can be found in the same hilar area (50, 113). However, it is unknown if the blood flow in TLS is connected to the larger vessels. Little is known about the lymphatic flow within TLS (114). There is also limited research on the role of certain immune cell types, such as Treg cells, within TLS.

The presence of TLS may also pose challenges to treatment in LN. For example, local B cells in TLS may not be easily inhibited by systemic therapy, such as rituximab. In a study of chronic renal allograft rejection, systemic rituximab failed to cause TLS regression in kidney despite the depletion of peripheral B cells (115). This finding suggests that the local TLS may facilitate B cell survival and allow the evasion of systemic rituximab-mediated depletion. A case series reported three cases with recurrent and refractory oral pemphigus vulgaris. Two of the three had negative circulating desmoglein antibodies, whereas intralesional rituximab was effective in the treatment of the two (116). Intralesional rituximab likely killed the lesional B cells in the TLS and enhanced the lesion healing. The presence of TLS may also explain the relapse and treatment resistance of local lesions in the absence of circulating autoantibodies.

## 8 Conclusions

TLS recapitulate the functions of SLO at local chronic inflammatory sites. After the initial loss of tolerance, the activated autoimmune cells may infiltrate the target organs, where they may induce the formation of TLS that, in turn, may maintain the autoimmune response by facilitating interactions among different immune cell types and between various macromolecules and immune cells. TLS may facilitate a local activation of T and B cells in tissues resulting in a rapid response to local antigens, which may resemble the systemic adaptive immune response that occurs in SLO. Additionally, TLS may provide a platform for encounters between local autoantigens, antigen-presenting cells and lymphocytes, thus promoting an efficient antigen processing and presentation leading to the spreading of autoimmune response to additional autoantigens. In fact, animal studies have demonstrated the induction of high affinity antibodies in TLS. Furthermore, locally activated immune cells as well as tissue-resident cells may become a source of inflammatory mediators, thus exacerbating kidney injury. Both animal model and human studies support the role of TLS in the kidney to amplify autoimmune responses as well as cause the progression of renal damage in LN. These observations provide a strong rationale for developing new therapies that target events that initiate TLS formation, molecules that promote the maturation and organization of TLS, and by depleting the locally activated immune cells in the TLS of kidneys in LN. However, there are still many hurdles to resolve before realizing the potential of anti-TLS therapies. For example, the lack of non-invasive methods to visualize/quantify TLS in kidneys is a major hurdle in using TLS for clinical assessment, but recent success in visualizing TLS in the pancreas of lupus-prone mice by SPECT provides hope for early detection of TLS in LN using *in vivo* imaging.

## Author contributions

MW and RS developed the outline and objective of the article, MW and YL wrote the draft of the article, TI and DN assisted with literature review, preparation of figures, and writing, and MW and RS revised it critically for important intellectual content. XL, SR, and JH contributed to the review. All authors contributed to the article and approved the submitted version.
